# Phytanic acid activates NADPH oxidase through transactivation of epidermal growth factor receptor in vascular smooth muscle cells

**DOI:** 10.1186/s12944-016-0273-9

**Published:** 2016-06-10

**Authors:** Gursev S. Dhaunsi, Mayra Alsaeid, Saghir Akhtar

**Affiliations:** Departments of Pediatrics, Faculty of Medicine, Kuwait University, Kuwait City, Kuwait; Pharmacology and Toxicology, Faculty of Medicine, Kuwait University, Kuwait City, Kuwait

**Keywords:** Phytanic acid, Aortic smooth muscle, NADPH oxidase, EGFR

## Abstract

**Background:**

Phytanic acid (PA) has been implicated in development of cancer and its defective metabolism is known to cause life-threatening conditions, such as Refsum disease, in children. To explore molecular mechanisms of phytanic acid-induced cellular pathology, we investigated its effect on NADPH oxidase (NOX) and epidermal growth factor receptor (EGFR) in rat aortic smooth muscle cells (RASMC).

**Methods:**

Smooth muscle cells were isolated from rat aortae using enzymic digestion with collagenase and elastase. Cultured RASMC were treated with varying concentrations (0.5-10 μg/ml) of phytanic acid in the presence/absence of fetal bovine serum (FBS) and/or EGFR inhibitor, AG1478. Following treatment with experimental agents, NOX activity was assayed in RASMC cultures by luminescence method. Gene expression of NOX-1 and p47phox was assessed using RT-PCR. NOX-1, p47phox and, total EGFR protein and its phosphorylated form were measured by Western blotting.

**Results:**

Treatment of RASMC with supraphysiological concentrations (>2.5 μg/ml) of PA significantly (*p <* 0.01) increased the NOX activity. PA also significantly increased gene/protein expression of NOX-1 and p47phox whereas p22phox and p67phox remained unaffected. Interestingly, PA (2.5-10 μg/ml) markedly (2–3 folds) increased the total and phosphorylated EGFR. Treatment of cells with EGFR inhibitor, AG1478, significantly blocked the PA-induced enhancement of NOX activity.

**Conclusions:**

Our findings that PA transactivates EGFR and induces NOX activity in vascular smooth muscle cells provide new insights into molecular mechanisms of PA’s role in cancer and Refsum disease.

## Background

Phytanic acid (PA) is a branched fatty acid that is synthesized from phytol during degradation of plant chlorophyll and catabolized in mammalian cells through peroxisome enzyme system [1, 2]. Major amount of circulating phytanic acid in humans comes from dietary sources such as meats and dairy products [3]. PA metabolism was recognized to be vital for human health with the identification of peroxisomal disorders, such as Zellweger syndrome and Refsum disease where supra-physiological amounts of phytanic acid were found to accumulate in body tissues and fluids of the patients [4–6]. Peroxisomal disorder patients with aberrant phytanic acid metabolism often experience severe clinical complications that range neurological impairment to cardiovascular anomalies [7, 8]. PA has been reported to inhibit Na^+^, K^+^-ATPase activity and mitochondrial respiratory chain complex (s) possibly causing impairment of synaptic function [9, 10]. A number of nuclear transcription factors called peroxisome proliferator-activated receptors (PPAR), particularly PPAR-α, have strong affinity for PA and their activation through ligand binding affects lipid metabolism, besides other responses [11]. Idel and co-workers [12] have reported that supraphysiological levels of phytanic acid induce nitric oxide-mediated apoptosis in cultured vascular smooth muscle cells suggesting thereby that phytanic acid might have a role in regulation of cell growth in vivo. Though nitric oxide has recently been implicated in phytanic acid-induced apoptosis of smooth muscle cells, any role of reactive oxygen species such as highly reactive superoxide anion production in relation to phytanic acid-mediated regulation of vascular growth remains to be examined. NADPH oxidase (NOX), a multicomponent enzyme system, is a major source of superoxide anion formation in various tissues including vascular smooth muscle cells [13, 14]. Though originally reported for its presence in phagocytes, NOX is now known to be expressed in all vascular cell types and participates in various physiological functions such as regulation of vascular tone and pathological conditions such as diabetes, hypertension and atherosclerosis [15–17]. In vascular smooth muscle cells, NOX activity is regulated by a catalytic unit NOX-1 and several subunits such as p22phox, p47phox, p67phox and rac-1, and one or more than one of these components of NOX have been reported to be modulated during different pathological conditions [18, 19]. Though NOX system has been extensively investigated and reported for its modulation by various vasoactive molecules such as angiotensin-II, PDGF and cytokines [20–22], it has remained unclear if phytanic acid, a biomolecule linked with severe cellular pathology in peroxisomal disease, has any influence on superoxide anion production by NOX.

Recently, increased serum levels of PA have been linked to development of several types of cancers that include prostate, breast and colon [23], however molecular mechanism (s) of PA-induced cellular pathology in carcinogenesis remain unknown. Overproduction of reactive oxygen species (ROS) has been reported as one of the several culprits for development of cancer in humans and NOX-mediated generation of ROS is known to contribute towards formation of tumors [24, 25]. EGFR, a cell surface receptor with intrinsic protein kinase activity, has been recognized as a key player in vascular biology and, development and progression of cancer due to its diverse signaling responses to regulate cellular proliferation, differentiation, migration and survival [26, 27]. EGFR mutations and overexpression have been widely linked to various types of cancers, leading the way to development of EGFR inhibitors as anticancer agents [28]. PA-mediated risk of cancer has attracted the attention of several research groups, yet any role of EGFR, a key cell growth regulator, in PA-induced cellular pathology has remained unexplored. This study was carried out to investigate the effect of PA on NOX and EGFR in vascular smooth muscle cells, to understand cellular and molecular mechanisms of PA-induced pathogenesis in peroxisomal disorders and development of cancer. Vascular smooth muscle cells have been used in previous studies to examine PA-induced pathogenic effects. Vascular smooth muscles were employed as an experimental model in this study due to their well reported active participation in lipid metabolism and their role in growth factors-/receptors- and NOX-mediated pathogenesis in proliferative vascular diseases.

## Methods

Male Wistar Rats (weighing 100-125 g) were used in this study according to the United States National Institute of Health (NIH) guidelines for the Care and Use of Laboratory Animals (NIH Publication No. 85–23, revised in 1996). The study protocols (MK01/12) were approved by the Research Ethics Committee of Health Sciences Center, Kuwait University.

### Materials

Bovine serum albumin (BSA), penicillin/streptomycin and fetal bovine serum (FBS) were purchased from Sigma Chemical Company (St. Louis, MO). DMEM-Ham’s F-12 (1:1) and trypsin-EDTA were from GIBCO (Grand Island, NY). Lucigenin, NADPH and phytanic acid were purchased from Sigma Chemical Co. Primaria tissue culture plates were obtained from Falcon Becton Dickinson (Oxnard, CA). All other reagents were of highest quality available and purchased from Sigma or Calbiochem.

### Methods

#### Aortic smooth muscle cell cultures

Rats were anesthetized with metofane and sacrificed by ventricular puncture for the removal of thoracic aortae. Aortic smooth muscle cells were cultured using enzymic digestion of aortic tissue by collagenase and elastase as described earlier [29]. Thoracic aortas were cleaned of the adherent fatty tissue and washed with sterile Hank’s medium. Aortas were then incubated for 20 min at 37 ° C in the digestion mixture that contained 1.5 mg/ml BSA, 25U/ml of pancreatic Elastase (Sigma, USA) and 200U/ml Collagenase (type IX, Sigma, USA). After the incubation period, adventitia was removed and, the medial layer was cut into small fragments and digested by incubation in digestion mixture for another 45 min followed by washing twice with fresh DMEM and centrifugation. Isolated cells were suspended in DMEM-F12 HAM containing 10 % heat-inactivated fetal bovine serum and plated onto 25-cm^2^ culture flasks for culture in humidified conditions under 5 % CO_2_. The obtained RASMC were characterized by immunostaining with monoclonal antibody specific for smooth muscle α-actin.

#### Treatment of cell cultures

Aortic smooth muscle cells grown in culture plates were used in all experiments. Before adding the experimental agents, cell monolayers were washed twice with serum free DMEM-F/12 medium (SFM) and incubated at 37 °C in a humidified cell culture incubator for 2 h in the presence of 0.1 % FBS containing DMEM-F/12 medium. Phytanic acid (0–10 μg/ml) was mixed with α-cyclodextrin-containing DMSO (0.001 % v/v) and added to the cell cultures in the presence or absence of FBS (5 %) and/or 50 μM of AG1478. Following the addition of experimental agents, cells were incubated in cell culture incubator for another 20–24 h at 37 °C.

#### Assay of NADPH oxidase activity

NADPH oxidase activity was measured in cell homogenates at 37 °C using lucigenin and NADPH as described elsewhere [30]. Briefly, NOX activity was measured in cell homogenates in a reaction mixture that contained 50 mM phosphate buffer, pH 7.1, 0.01 mM EDTA and 25 μM lucigenin. Reaction was started by addition of 100 μM of NADPH and chemluminiscence was recorded over a period of 3 min. Specific enzyme activity was calculated as relative light units (RLU) emitted per sec per mg of protein.

#### RNA isolation and reverse transcription

In each experiment, total RNA was extracted from cultured smooth muscle cells with RNA extraction kit based on use of guanidinium thiocyanate, lithium chloride and cesium triflouroacetate. Isolated RNA was of high quality and was used immediately for synthesis of first strand cDNA according to protocols from Clonetech’s SMART PCR cDNA synthesis kit.

#### PCR detection of mRNA for NOX-1, p22phox, p47phox, p67phox and G3PDH

Amplification of cDNA obtained from reverse transcription of RNAs from RASMCs was carried out using Advantage cDNA PCR kit (BD Biosciences Clonetech) and the following primers: NOX-1; 5′-GCC AGA CTC AGA GTT GGA GAT GCT–3′ and 5′-GCA GTT TCA AGA TGC GTG GAA ACT A-3′, p22phox; 5′-GTA GAT GCC GCT CGC AAT GGC CAG-3′ and 5′- ATG GGG CAG ATC GAG TGG GCC ATG T-3′, p47phox; 5′-CTT TGG GCA TCA AGT ATG TCT C-3′ and 5′-ATC AAT CCA GAG AAC AGG ATC A-3′ and p67phox; 5′-TGC CTT TTC CAG TAC TAC CTA TGT C-3′ and 5′-CTC TCA TCT GAC ACT CCC ATT TAA C-3′. Primers for G3PDH were provided by Clonetech. First strand of cDNA obtained from reverse transcription was denatured for 1 min at 95 °C and subjected to PCR with following parameters; 95 °C for 30 sec, 58 °C or 62 °C for 30 sec, 68 °C for 45 sec, 25–30 cycles after denaturing at 95 °C for 1 min. PCR products for various NOX components were analyzed using 2 % agarose gel electrophoresis.

### Western blot analysis of NOX-1, p47 phox and EGFR

Western blotting for NOX-1, p47 phox and total or phosphorylated forms of EGFR was performed as described earlier [31]. Briefly, cell pellets were transferred to lysis buffer (pH 7.6) containing 50 mM Tris-base, 5 mM EGTA, 150 mM NaCl, 1 % Triton 100, 2 mM Na_3_VO_4_, 50 mM NAF, 1 mM PMSF, 20 μM phenylarsine, 10 mM sodium molybdate, 10 μg/ml leupeptin and 8 μg/ml aprotinin). Aliquots containing equal amounts of protein were subjected to SDS-PAGE gel electrophoresis (SDS-PAGE) and transferred onto nitrocellulose membrane. Monoclonal antibodies were used to detect NOX-1, p47 phox and, phosphorylated and total forms of EGFR. Secondary antibodies conjugated to horseradish peroxidase were employed to get immunoreactive bands that were detected with Super Signal chemiluminescent substrate. Images were finally analyzed and quantified by densitometry and all data normalized to β-actin levels.

### Data analysis

Values shown in the results are mean ± standard deviation of four experiments carried out in triplicate. Student’s t test was employed using SPSS software to determine statistical significance of the results.

## Results

### Effect of Phytanic acid on NOX in RASMC cultures

**Fig. 1:**
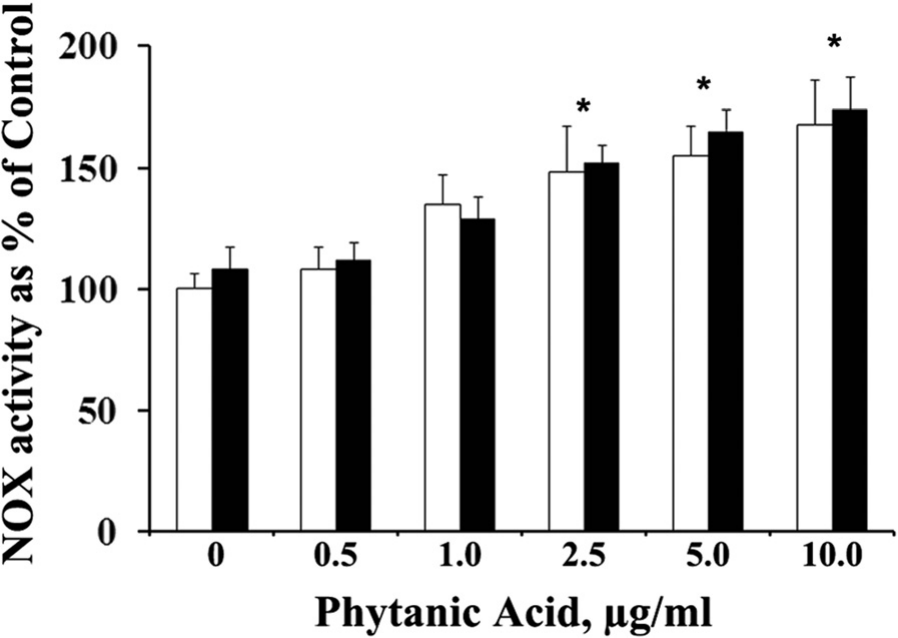
NADPH oxidase activity (shown as percent of control) in cultured RASMC following treatment with 0–10 μg/ml of phytanic acid in the presence (dark bars) or absence (light bars) of 5 % FBS. Values are Mean ± SD of at least six measurements. *p < 0.01 when compared with control (0 μg/ml of phytanic acid with or without FBS)

**Fig. 2:**
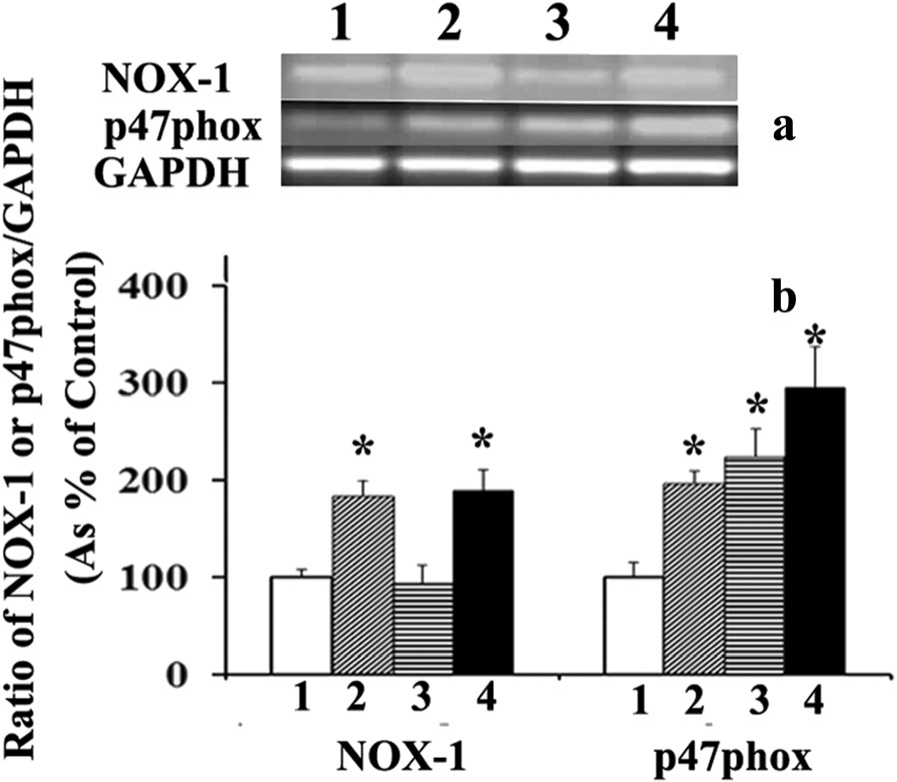
Gene expression of NOX-1 and p47phox in RASMC cultured in serum free medium (lane 1) or 5 μg/ml of phytanic acid (lane 2) or 5 % FBS (lane 3) or FBS and phytanic acid (lane 4). Panel A shows the representative bands for mRNA levels of NOX-1, p47 phox and GAPDH. Panel B shows the percent ratio of NOX/GAPDH or p47phox/GAPDH in RASMC. Values are Mean ± SD of at least six measurements. *p < 0.01 when compared with control (without FBS or phytanic acid)

**Fig. 3:**
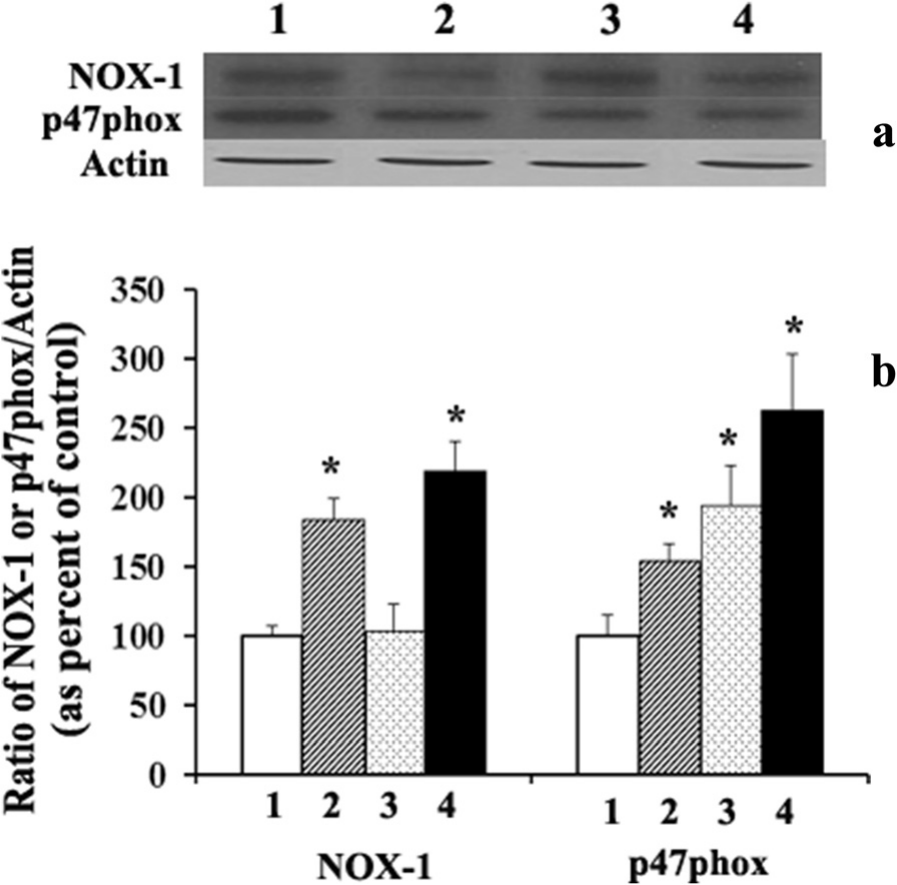
Western blot analysis of NOX-1 and p47phox in RASMC cultures treated with serum free medium (lane 1) or 5 μg/ml of phytanic acid (lane 2) or 5 % FBS (lane 3) or FBS and phytanic acid (lane 4). Panel A shows the representative protein bands of NOX-1, p47 phox and actin. Panel B shows the ratio of NOX/actin or p47phox/actin in RASMC. Values are Mean ± SD of at least six measurements. *p < 0.01 when compared with control (lane 1)

Figure 1 shows that 24 h treatment of RASMC with supra-physiological concentrations (2.5-10 μg/ml) of phytanic acid significantly (*p <* 0.01) increased the NOX activity irrespective of the presence or absence of FBS in the culture medium. In relation to the observed activation of NOX activity by phytanic acid, we examined the gene/protein expression and of various NOX components in RASMC cultures treated with phytanic acid in the presence or absence of FBS. Figure 2 shows the results of PCR analysis of NOX-1, the catalytic unit of NOX and sub-component p47phox in smooth muscle cells following treatment with PA and or FBS. PCR analysis illustrated in Fig. 2b shows that the ratios of the levels of NOX-1 or p47phox gene transcripts and GAPDH were significantly increased (*p <*0.01) in RASMC treated with phytanic acid (5 μg/ml) in the presence or absence of 5 % FBS. Stimulation of cells with FBS–alone did not have any significant effect on the gene transcripts of NOX-1, however p47phox was significantly (*p <* 0.01) elevated. Protein levels of NOX-1 as well as p47phox were markedly increased following treatment of RASMC with phytanic acid in the presence or absence of FBS (Fig. 3). Phytanic acid treatment, however did not have any significant effect on gene expression of p22phox or p67phox in RASMC cultures (data not shown).

### Effect of phytanic acid on EGFR

**Fig. 4:**
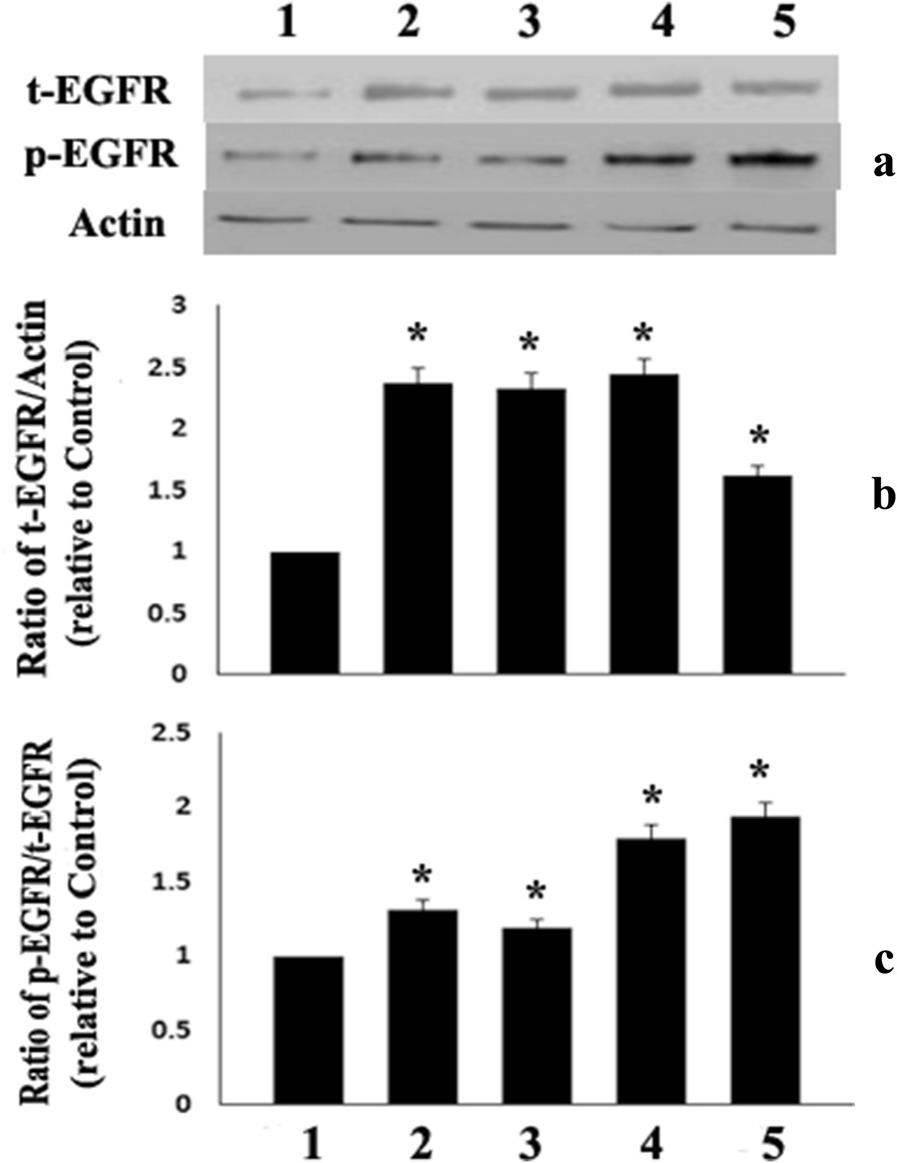
Panel **a** illustrates representative western blots of total EGFR protein (t-EGFR), phosphorylated EGFR (p-EGFR) and Actin in RASMC cultures. Panel **b** shows the levels of t-EGFR and panel C illustrates the ratio of p-EGFR/t-EGFR in RASMC treated with 2.5 μg/ml (lane 2), 5.0 μg/ml (lane 3) or 10 μg/ml (lane 4) of Phytanic acid. Lane 1 represents RASMC without any treatment whereas lane 5 had RASMC treated with 10 μg/ml of PA and 5 % FBS. Values are Mean ± SD of six measurements. * *p <* 0.1 when compared with control (lane 1)

Figure 4 shows that treatment of RASMC with supraphysiological concentrations (2.5 -10 μg/ml) of phytanic acid significantly (*p <* 0.01) increased the expression of total EGFR protein in the presence or absence of FBS. Phytanic acid also significantly (*p <* 0.01) enhanced the phosphorylation of EGFR, however the effect was more pronounced in cells treated with 10 μg/ml of PA in the presence of FBS.

### Effect of AG1478 on PA-induced NOX activity

**Fig. 5:**
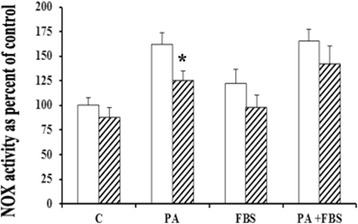
NOX activity in RASMC cultures following treatment with phytanic acid (10 μg/ml) and/or FBS (5 %) in the absence (open bars) or presence (shaded bars) of EGFR inhibitor, AG1478 (50 μM). Values of NOX activity shown as percent of Control are Mean ± SD of six measurements. * *p <* 0.01 when compared with Control (without FBS and PA)

EGFR specific inhibitor, AG1478, significantly (*p <* 0.01) blocked the PA-induced NOX activity as shown in Fig. 5. FBS did not have any significant effect on NOX activity in the presence or absence of PA and/or AG1478.

## Discussion

A marked increase in the levels of phytanic acid in body tissues and fluids of patients with Refsum disease and Zellweger syndrome has, for many years, implicated this branched fatty acid in development and progression of the disease [4, 5], yet the underlying molecular mechanisms of such a role for PA in pathogenesis of peroxisomal diseases have remained elusive. More recently, increased levels of PA have been linked with an increased risk of developing cancer [25], however pathogenic mechanisms have remained unidentified. This study provides some new insights into molecular mechanisms of PA-mediated cellular pathology.

Besides several other mechanisms suggested for the regulation of cellular functions by different fatty acids, intracellular production of or exposure to extracellular cytokines, reactive oxygen species and nitric oxide production have been reported to mediate fatty acid–induced effects on cell survival [32, 33]. The supraphysiological concentrations of phytanic acid have been shown earlier to enhance nitric oxide production in vascular smooth muscle cells and induce apoptosis [12]. Nitric oxide is an important regulator of vascular biology and has been widely reported to inhibit vascular growth in vivo as well as in cell culture studies [34]. Nitric oxide-mediated effects have been reported to be influenced by the superoxide anion production, as these two species interact to generate a highly reactive peroxynitrite molecule [35]. Our findings that PA activates NOX activity through upregulation of NOX-1 and p47phox proteins, strongly suggests that nitric oxide may not be the only reactive nitrogen/oxygen species formed in response to phytanic acid, superoxide formation as a result of NOX activation, may well be a key ROS produced in response to PA that may further lead to formation of highly reactive peroxynitrite. ROS, such as superoxide radicals and hydrogen peroxide have been reported earlier to modulate the proliferation of smooth muscle cells in vivo as well as in vitro studies [36]. Enzymatic activity of NOX has been well reported to be regulated through interaction of its various components, p22phox, p47phox and p67phox. Our findings that phytanic acid enhances NOX-1 and p47phox levels through transcriptional and translational activation without having any significant effect on expression of p22 phox and p67phox proteins indicate that this branched fatty acid possibly plays a role in regulation of cellular oxidative stress through activation of NOX and thus provides a novel molecular mechanism for the actions of phytanic acid in conditions of peroxisomal dysfunction or carcinogenesis. Our finding that PA-induced enhancement of NOX activity is partly blocked by AG1478, an EGFR inhibitor, unravels the involvement of EGFR, another key cell growth regulator, in PA-mediated pathogenic events. Over expression of EGFR is associated with cancer development. The fact that PA increased the expression of EGFR protein and enhanced its phosphorylation strongly indicates that the reported carcinogenic capability of PA in various types of cancers is likely occurring through activation of EGFR tyrosine kinase.

Impairment of the PA-induced NOX activity by EGFR inhibitor strongly suggests that induction of EGFR gene expression and phosphorylation of EGFR precede the activation of NOX activity in response to ex vivo treatment of RASMC with PA. Future in vivo studies might provide physiological importance of our findings however, this ex vivo study on RASMC cultures establishes an important link between PA-induced transactivation of EGFR and formation of NOX-mediated ROS production providing a possible molecular mechanism of cellular pathology of peroxisomal dysfunction in cases of Refsum disease and PA-related carcinogenesis.

## Conclusions

This ex vivo study carried out on vascular smooth muscle cells concludes that PA increases  NOX activity through transcriptional activation of NOX-1 and p47phox. An increase in phosphorylation of EGFR by PA and attenuation of PA-induced NOX activity by EGFR inhibitor provide an important insight into molecular mechanism of PA's role in pathogenesis of some peroxisomal diseases and cancers.
